# China nationwide landscape of 16 types inherited metabolic disorders: a retrospective analysis on 372,255 clinical cases

**DOI:** 10.1186/s13023-023-02834-y

**Published:** 2023-08-03

**Authors:** Beibei Zhao, Peichun Chen, Xuhui She, Xiuru Chen, Zhou Ni, Duo Zhou, Zinan Yu, Chang Liu, Xinwen Huang

**Affiliations:** 1 Department of Genetics and Metabolism, Children’s Hospital of Zhejiang University School of Medicine, National Clinical Research Center for Child Health, 3333 Bisheng Road, Hangzhou City, 310052 Zhejiang Province China; 2 Clinical Mass Spectrometry Center, Guangzhou KingMed Center for Clinical Laboratory Co., Ltd., Guangzhou International Bioisland, No.10 Luoxuan Third Road, Guangzhou City, 510005 Guangdong Province China; 3 Guangdong Provincial Key Laboratory of Genetic Disease Diagnositc, Guangzhou International Bioisand, No.10 Luoxuan Third Road, Guangzhou City, 510005 Guangdong Province China; 4 Shenzhen Guangming Maternity and Child Healthcare Hospital, University of Chinese Academy of Science, No.39 of Huaxia Road, Guangming District, Shenzhen, 518107 Guangdong China

**Keywords:** Inherited metabolic disorders, Organic acids, Amino acids and acylcarnitines, Incidence and regional distributions, China

## Abstract

**Background:**

Inherited metabolic disorders (IMDs) usually occurs at young age and hence it severely threatening the health and life of young people. While so far there lacks a comprehensive study which can reveals China’s nationwide landscape of IMDs. This study aimed to evaluate IMDs incidence and regional distributions in China at a national and province level to guide clinicians and policy makers.

**Methods:**

The retrospective study conducted from January 2012 to March 2021, we analyzed and characterized 372255 cases’ clinical test information and diagnostic data from KingMed Diagnostics Laboratory. The samples were from 32 provincial regions of China, the urine organic acids were detected by gas chromatography-mass spectrometry (GC–MS), amino acids and acylcarnitines in dried blood spots were detected by liquid chromatography-tandem mass spectrometry (LC–MS/MS). We did a statistical analysis of the distribution of the 16 most common IMDs in amino acid disorders and organic acidemias, and then paid special attention to analyze the age and regional distributions of different IMDs. The statistical analyses and visualization analysis were performed with the programming language R (version 4.2.1).

**Results:**

There were 4911 positive cases diagnosed, which was 1.32% of the total sample during the ten-year study period. Most diseases tended to occur at ages younger than 18 year-old. The Ornithine Transcarbamylase Deficiency tended to progress on male infants who were less than 28 days old. While the peak of the positive case number of Citrin Deficiency disease (CD) was at 1–6 months. Different IMDs’ had different distribution patterns in China’s provinces. Methylmalonic Acidemias and Hyperphenylalaninemia had an imbalanced distribution pattern in China and its positive rate was significantly higher in North China than South China. Conversely, the positive rate of CD was significantly higher in South China than North China.

**Conclusions:**

Results of this work, such as the differences in distribution pattern of different diseases in terms of age, region, etc. provide important insights and references for clinicians, researchers and healthcare policy makers. The policy makers could optimize the better health screening programs for covering children and infants in specific ages and regions based on our findings.

**Supplementary Information:**

The online version contains supplementary material available at 10.1186/s13023-023-02834-y.

## Background

Inherited Metabolic Disorders (IMDs), which is also termed “Inborn Errors of Metabolism”, is a class of inborn genetic diseases. Usually, it begins with defects on genes encoding enzymes. Resultant defected enzymes fail to normally catalyze a variety of in vivo chemical reactions, causing metabolic malfunctions. Consequently, a large amount of substance may accumulate, causing toxic effects or malfunctions in vivo [1]. Decades ago, diagnosis of IMDs was difficult and many types of IMDs were untreatable. Nowadays, thanks to the advancement of technologies, diagnosis of IMDs is facilitated and improved by using mass spectrometry (MS)-based technologies [2]. And parts of previously untreatable IMDs now become treatable. Conventional therapies for several IMDs are dietary restriction, dietary supplementation, etc. Recently, advanced therapies such as gene therapy and enzyme replacement are available [3].

Urine organic acids could be used as specific diagnostic indicators for certain kinds of IMDs. E.g., a part of amino acid disorders and organic acidemias. And hence hospitals or clinical laboratories measure the concentrations of organic acids to indicate people’s conditions of relevant IMDs [4]. Currently, most hospitals and medical laboratories measure amino acids and acylcarnitines in dried blood spot samples for early screening of IMDs, including amino acid disorders, organic acidemia and fatty acid oxidation disorders [5]. While urine could also be a useful and reliable source for diagnosing several types of IMDs because concentrations of a variety of organic acids and other components could also be quantitated as well [6]. For example, using urine for clinical tests, the increase of homogentisic acid could indicate people’s conditions of Alkaptonuria (AKU, OMIM# 203,500). The increase of isovalerylglycine could indicate people’s conditions of Isovaleric Acidemia (IVA, OMIM# 243,500). The increase of Glutaric acid, Glutaconic acid, 3-hydroxyglutaric acid could indicate people’s conditions of Glutaric Acidemia Type I (GA-I, OMIM# 231,670). Specifically, using blood samples alone for clinical tests, it is difficult to distinguish between Methylmalonic Acidemias (MMA, OMIM# 251,000, 277,400, 277,410, 251,100, 251,110, 277,380, 309,541, 613,646, 614,265 and 614,857) and Propionic Acidemia (PA, OMIM# 606,054). While quantitation of methylmalonic acid and methylcitric acid from people’s urine samples could indicate people’s conditions of MMA [7–9]. Moreover, together with the measurement of concentration of citrulline in people’s blood sample, measuring concentrations of organic acids in people’s urine, e.g., 4-hydroxy phenyllactic acid, 4-hydroxy phenylpyruvic acid, orotic acid, and uracil, could indicate other disorders such as Citrullinemia Type I (CIT-I, OMIM# 215,700), Citrin Deficiency disease (CD, OMIM# 605,814 and 603,471) and Ornithine Transcarbamylase Deficiency (OTCD, OMIM# 311,250) [10]. Therefore metabolites in urine are also valuable for IMDs diagnosis.

Several studies have been conducted to estimate the prevalence of IMDs in preliminary screening and high-risk screening, as shown in Table 1. Epidemiological data of the above studies indicate that IMDs seemed have a relatively low prevalence in general compared with other more commonly seen diseases. Despite such data from above studies, IMDs are by no means less important than other diseases. Instead, IMDs should be paid high attention to because they are closely associated with early neonatal death and abnormal growth and development.

**Table 1:** Summary of published on prevalence of IMDs

Reference	Study population	Study factors	Diseases (number of types)	Clinical diagnostic testing	Samples	Period	Region	Rate (positive/total)
Wajner M, et al. [11]	Inpatients of hospitals from 10 different Brazilian states	Establish prevalence of organic acidopathies in a high-risk Brazilian population	Organic acidemias	Organic acid, enzyme determination, Gene mutation analysis, amino acid and acylcarnitines	Urine specimens, dried blood and plasma	January 1994 to July 2001	Brazil	4.8%(93:1926)
Tan IK, et al. [12]	Patients with symptoms suggestive of an IMDs	A nationwide study of IMDs	48 IMDs, including organic acidurias, amino acidaemias/acidurias, urea cycle defects, mucopolysaccharidoses, carbohydrate disorders and others	Amino acids, organic acids,mucopolysaccharides, sugars	Blood and/or urine	1992 to 2005	Singapore	3.5%(127:3656)
Han L, et al. [13]	Patients with suspected IMDs	Analyzed age distributions, prevalence, and age of onset for IMDs in Chinese patients	28 IMDs, including 12 amino acid disorders, 9 organic acidemias and 7 fatty acid oxidation disorders	Amino acids and acylcarnitines, organic acid, clinical features,conventional laboratory tests,enzyme activity tests,gene mutation analysis	Blood and/or urine	February 2002 to June 2012	China	6.2%(1135:18,303)
Hampe MH, et al. [14]	Indian children with demographic and clinical	Feasibility of GC–MS to detect IMDs in India	27 IMDs, including primary lactic acidemia, organic acidemia, amino acid disorders and others	Enzyme analysis, organic acids, amino acids, fatty acids, and sugars	Urine	July 2013 to January 2016	Indian	1.4%(323:23,140)
Lindner M, et al.[15]	90% NBS samples were sent from obstetric units or children’s hospitals and 10% from midwives or general paediatricians	Systematic evaluations of newborn screening programmes	36 IMDs, including 12 amino acid and Urea cycle disorders, 9 fatty acid oxidation disorders, 13 organic acidemias disorders and others	NBS and confirmatory diagnostics (including enzyme activity, informative genotype)	Blood and/or urine, fibroblasts, lymphocytes	January 1999 to June 2009	South-West Germany	0.034%(373:1,084,195)
Li X, et al.[16]	Newborns in Changsha, China	Evaluate the characteristics of IMDs in Changsha, China	41 kinds of IMDs, including amino acid disorders, organic acidemias and fatty acid oxidation disorders	NBS and organic acids, urinary pterin, dihydropteridine reductase, genetic analytic	Blood and/or urine	January 2016 to December 2020	Changsha,China	0.024%(71:300,849)
Tiivoja E, et al.[17]	Population in Estonia	Define the prevalence and live birth prevalence of IMDs and the effectiveness of new diagnostic methods on the diagnosis of IMD	Amino acid disorders, disoreders of complex molecule degradation, mitochondrial disorders, disorders of energy substrate metabolism, disorders of fatty acid and ketone body metabolism	Diagnostic algorithm, urine creatine&guanidinoacetate, acylcarnitine analysis, serum transferrin isoelectric focusing, exome sequencing& NGS panels	Blood and/or urine	1990 to 2017	Estonian	0.017%(333:1,919,133)

*NBS* Newbron Screening; *GC-MS* Gas chromotography coupled to mass spectrometry, *IMDs* Inherited Metabolic Disorders

However, to our best knowledge, there lacks such comprehensive analysis and up-to-date study, which can provide IMDs testing data of a large population and reflect the global IMDs landscape of a nation. For regional IMDs information, the aforementioned studies could be referred to some extent [11–17] (Table 1). While in terms of IMDs information covering greater areas, the above works had limited sample sizes, and hence have limited reference value for greater areas. For instance, Han et al. described an analysis on 18,303 patients’ organic acid assays and IMDs data collected from February 2002 to June 2012. Though this work covered most provinces of China, yet several provinces such as Henan, Tibet, and Inner Mongolia regions were not included, and thus it was not a nationwide study [13]. Therefore, the objective of the study is to evaluate IMDs incidence and regional distributions in China at a national and province level. To do so, we analyzed the age and regional distributions of different IMDs. This work revealed the latest IMDs information of both the provincial domestic situation and the nationwide situation in China, it will provide a reference for promoting policy implementation and resource allocation for IMDs.

## Methods

To report this study, the Strengthening the Reporting of Observational Studies in Epidemiology (STROBE) statement was used [18].

### Study aim and design

The study aims to evaluate IMDs incidence and regional distributions in China at a national and province level utilizing a retrospective study design. We comprehensively reviewed and analyzed a large dataset which included diagnostic information of 16 types of IMDs and other clinical feature information of 372,255 Chinese from different provinces of China. The 16 types of IMDs are shown in Table 2, including the most common diseases in amino acid disorders and organic acidemias.

**Table 2:** Summary of dataset

IMD	OMIM code	Positive case (percentage)	Positive case number of gender (Male:Female:Missing)	Rate/100,000 population	95% CI of the population
*Amino acid disorders*					
HPA	#261,600,#233,910,#261,640,#264,070,#261,630,#617,384	410 (8.35)	226:184:0	110.14	100.11; 121.46
CD	#603,471,#605,814	361 (7.35)	202:159:0	96.98	87.61; 107.64
OTCD	#311,250	190 (3.87)	129:61:0	51.04	44.41; 58.97
MUSD	#248,600	114 (2.32)	67:47:0	30.62	25.62; 36.93
CIT-I	#215,700	25 (0.51)	15:10:0	6.72	4.66; 10.07
AKU	#203,500	14 (0.29)	12:2:0	3.76	2.34; 6.48
TYR-I	#276,700	7 (0.14)	4:3:0	1.88	1.00; 4.06
*Organic acidemias*					
MMA	#251,000,#277,410,#277,400,#251,100,#251,110,#277,380,#309,541,#613,646,#614,265,#614,857	3046 (62.02)	1689:1351:6	818.26	789.95; 847.84
PA	#606,054	266 (5.42)	138:128:0	71.46	63.5; 80.71
GA-I	#231,670	248 (5.05)	126:122:0	66.62	58.96; 75.58
IVA	#243,500	111 (2.26)	66:45:0	29.82	24.89; 36.05
MCD	#253,260,#253,270	60 (1.22)	35:25:0	16.12	12.64; 20.9
3-MCCD	#210,200,#210,210	40 (0.81)	24:16:0	10.75	8.01; 14.79
MAD	#248,360	8 (0.16)	5:3:0	2.15	1.18; 4.42
MGA	#250,950	6 (0.12)	1:5:0	1.61	0.82; 3.7
HMGCLD	#246,450	5 (0.10)	3:1:1	1.34	0.66; 3.33
Sum		4911 (100.00)	2742:2162:7	1319.26	1283.23; 1356.55

HPA: Hyperphenylalaninemia; CD: Citrin Deficiency Disease; OTCD: Ornithine Transcarbamylase Deficiency; MSUD: Maple Syrup Urine Disease; CIT-I: Citrullinemia Type I; AKU: Alkaptonuria; TYR-I:Tyrosinemia type I; MMA: Methylmalonic Acidemias; PA: Propionic Acidemia; GA-I: Glutaric Acidemia Type I; IVA: Isovaleric Acidemia; MCD: Multiple Carboxylase Deficiency; 3-MCCD: 3-Methyl Crotonyl-CoA Carboxylase Deficiency; MAD: Malonic Acidemia Deficiency;MGA:3-Methylglutaconyl-CoA Hydratase Deficiency; HMGCLD: 3-Hydroxy-3-Methylglutary-CoA Lyase Deficiency

### Setting

The datasets of this study were retrieved from the Laboratory Information Management System (LIMS) of KingMed Diagnostics Laboratory (briefly called KingMed in later description). We queried the records of those whose sample were sent to KingMed during January 2012 to March 2021. The repeated measurement cases are screened out through the LIMS, the repeated measurement data were excluded after manual verification, for the positive cases, only the results of the first positive diagnosis were retained, for cases with abnormal results requiring repeated measurement, only the first results will be retained. After exclusion of a part of invalid data and records, we lastly obtained valid records and data of 372,255 people. Specifically, we did not find any sample data from Hong Kong and Taiwan. Therefore in this work, we considered the sample number from these two areas to be zero.

### Participants

From 3705 hospitals located in different regions of China, information and sample of people who were suspected to have IMDs were sent to KingMed for clinical tests. Dried urine filter paper and dried blood spot samples from people suspected of IMDs were sent to KingMed under refrigerated conditions within 48 h, and then urine organic acid metabolites were detected and quantitated using the gas chromatography-mass spectrometry (GC–MS) method. Amino acids and acylcarnitines in blood spots were detected and quantitated using the liquid chromatography-tandem mass spectrometry (LC–MS/MS) method. These clinical test results were diagnosed by IMDs expert clinicians. For some patients, diagnosis was confirmed by enzyme activity tests and gene mutation tests. All above resultant dataset including diagnostic information and clinical information were stored in the databases of KingMed. In these assay records, 116 types of organic acids and 55 types of amino acids and acylcarnitines are considered relevant to IMDs (Additional file 1: Table s1). All diagnostic indicators associated with relevant IMDs were summarized in Additional file 2: Table s2. The 16 types of IMDs could be divided into two subcategories. i.e., amino acid disorders and organic acidemias (Table 1).

### Laboratory tests

#### Measurements of urine organic acid metabolites

The samples were treated as described by Kimura et al. [19], with some modifications: take urine equivalent to 0.2 mg creatinine, which was eluted using distilled water from dried urine filter paper, then incubated with 20 µL urease at 37℃ for 30 min to remove urea followed by the addition of 40 µL of internal standard (heptadecanoic acid, eicosanoic acid, and tropic acid). The mixed solution was alkalinized with 400 µL of saturated NaOH. And then 1 mL of 25 g/L hydroxylamine hydrochloride was added for oximation of keto groups. Such modifications could raise the performance for the diagnosis of MSUD and TYR-I [20]. The solution was incubated at room temperature for 60 min. The mixed solution was adjusted to pH 1–2 with 6 M HCl, and then extracted with 6 mL of ethyl acetate. The supernatant was evaporated to dryness, the residue was further derivatized by BSTFA/TMCS for 30 min at 80℃ and subsequent analysis of the derivatized extract was done by QP-2010 Ultra GC–MS (SHIMADZU, Kyoto, Japan) [21].

#### Measurements of amino acids and acylcarnitines in dried blood spots

LC–MS/MS was used to detect the concentration of amino acids and acylcarnitines in dried blood spots. The sample preparation followed the derivatization method reported by Han et al [22]. The tandem mass spectrometers were API 3200/API 3200MD (SCIEX, Framingham, MA, USA), the high-performance liquid chromatograph system was a SHIMADZU LC-20AD.

### Statistical analysis

We characterized differences of IMDs’ distribution patterns by region, gender, age etc. Statistical analyses and graphs were performed using R version 4.2.1 [23]. Comparisons between the positive rate data of MMA and PA in different provinces in China were analyzed using the Student's test. A p-value of 0.05 or less was considered statistically significant and all tests were two-sided. 95% confidence interval is presented as Wilson score interval. Specifically, visualizations of positive case rates of different provinces were done using Package ‘highcharter’ (https://github.com/jbkunst/highcharter). Notably, in China, special administrative regions, municipalities and autonomous regions are in the same political class to provinces, such as Tibet autonomous region, Beijing municipality, Tianjin municipality, Chongqing municipality, and Macao special administrative region. So in this work, for simplicity, we also used the word "province" to refer to other special administrative regions, municipalities and autonomous regions. So, totally, in this work, 32 provinces of China were covered.

## Results

### Overview of dataset

Totally, our dataset included 372,255 cases of urine organic acid assay records. Out of these samples, 4911 (1.32%) patients were diagnosed with IMDs. Among these positive cases, we identified 10 pedigrees, of which 8 were found in MMA (6 pairs of twin sisters, 1 pair of brothers and 1 pair of fraternal twins), 1 pair of sisters in HPA, and 1 pair of siblings in GA-1. And these tests did not involve consanguineous marriage among the positive cases. Within the dataset, 218,123 cases are assay records of males and the 144,309 cases are assay records of females (for the rest 9793 assay records, the gender information is missing).

Compared with IMDs positive samples, the IMDs negative samples were the majority. In total of 372,255 samples, the number of negative one was 367,344 which was 98.68% of the total sample. Table 2 displayed the number of positive cases of 16 types of IMDs, the ratio of genders of positive cases of 16 IMDs. For males, 16 types of IMDs altogether had a positive rate of 0.74%. For females, that rate was 0.58%. Generally, the number of positive cases of most IMDs listed were not high compared to the total case number. Amongst 16 types of IMDs included in this work, MMA has the highest number of positive cases—3046, of which 1689 positive cases were male, 1351 positive cases were female, and the gender information of 6 cases is missing (Table 2). The number of 3046 accounts for 81.83 ‱ of the total sample number 372,255, and is obviously far beyond the positive case numbers of other IMDs. A quick look at gender comparison also revealed that, in general, male had higher number of positive cases than female in 15 types of IMDs listed, either slightly higher or significantly higher. Only for MGA, the number of positive cases of females was higher than that of males.

**Fig. 1:**
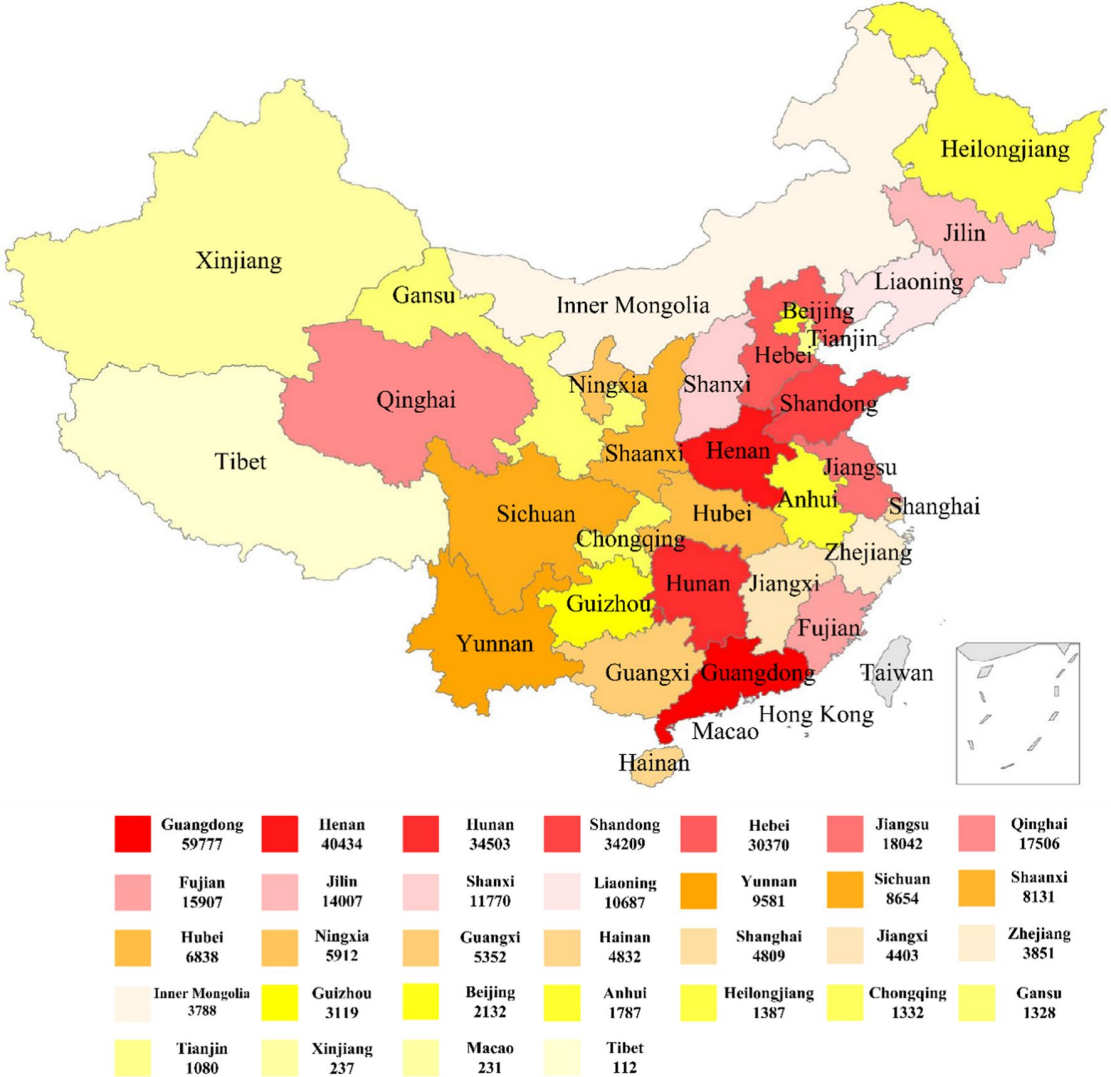
372,255 clinical samples’ distribution map of China provinces

Figure 1 displayed the China provincial distribution of provincial sample numbers of our dataset. According to the sum of the sample number of each province, the area of different provinces of China was colored with different intensities (Note that in all 372,255 cases, 6147 cases’ provincial information was missing). Amongst, the top-rank 5 provinces with the highest number of samples were Guangdong province, Henan province, Hunan province, Shangdong province, and Hebei province. Their sample numbers were 59,777, 40,434, 34,503, 34,209, and 30,370, respectively. We also noticed that, top-ranked 3 regions with the lowest number of sample were Tibet, Macao and Xinjiang. They had sample number of 112, 231, and 237, respectively. These numbers were quite low and had large differences compared with that of Tianjin. Tianjin had a sample number of 1080 and was the 4^th^ region with the least sample number.

### IMDs characteristics by ages

Table 3 displayed the age distribution of the assay records. We divided the age into 5 stages, with the earliest stage being for infants that were born less than (or equal to) 28 days. Most diseases tended to occur at age younger than 18 year-old. For example, the peak of positive case number of HPA was found at 7 to 36 month-old, which was also the peak for GA-I and MCD. While the peak of the positive case number of CD, MMA, 3-MCCD, and MGA was at 1–6 months. But an exception was MMA. Interestingly, though the peak of positive case number of MMA was found at 1–6 months, at ages older than 18 year-old where other IMDs had lower number of positive cases, MMA had the highest positive case number of 191 in contrast (Table 3).

**Table 3:** Age distribution of IMDs

IMDs	Positive case #	Age															
		≤ 28 days(n = 105,922)			1–6 M.O.^a^(n = 83,714)			7–36 M.O.^a^(n = 98,305)			4–18 Y.O.^b^(n = 72,129)			> 18 Y.O.^b^(n = 8352)			N.A.^c^(n = 3833)
		n	Rate/100,000 population	95% CI of the population	n	Rate/100,000 population	95% CI of the population	n	Rate/100,000 population	95% CI of the population	n	Rate/100,000 population	95% CI of the population	n	Rate/100,000 population	95% CI of the population	N.A.^c^
HPA	410	56	52.87	41.13;69.17	96	114.68	94.46;140.66	138	140.38	119.31;166.36	102	141.41	117.14;172.38	15	179.60	113.39;303.54	3
CD	361	13	12.27	7.52;21.59	329	393.00	353.4;438.35	15	15.26	9.63;25.81	0	0.00	0;0	1	11.97	4.15;77.73	3
OTCD	190	66	62.31	49.4;79.79	7	8.36	4.44;18.06	64	65.10	51.44;83.69	48	66.55	50.8;89	3	35.92	15.34;114.4	2
MSUD	114	67	63.25	50.23;80.84	21	25.09	16.88;39.07	17	17.29	11.19;28.32	7	9.70	5.16;20.96	0	0.00	0;0	2
CIT-I	25	10	9.44	5.46;17.99	3	3.58	1.53;11.42	8	8.14	4.47;16.73	4	5.55	2.55;15.25	0	0.00	0;0	0
AKU	14	2	1.89	0.73;7.62	3	3.58	1.53;11.42	3	3.05	1.3;9.72	5	6.93	3.38;17.19	0	0.00	0;0	1
TYR-I	7	1	0.94	0.33;6.13	1	1.19	0.41;7.76	4	4.07	1.87;11.19	1	1.39	0.48;9	0	0.00	0;0	0
MMA	3046	729	688.24	640.66;740.33	827	987.89	923.67;1057.77	756	769.04	716.8;826.12	484	671.02	614.66;734	191	2286.88	1992.86;2636.19	59
PA	266	91	85.91	70.41;105.98	41	48.98	36.61;67.12	94	95.62	78.61;117.55	36	49.91	36.64;69.89	1	11.97	4.15;77.73	3
GA-I	248	15	14.16	8.94;23.95	58	69.28	54.12;90.22	130	132.24	111.85;157.54	33	45.75	33.16;65.05	5	59.87	29.2;148.4	7
IVA	111	54	50.98	39.49;67.04	10	11.95	6.91;22.76	23	23.40	16;35.72	22	30.50	20.7;47.01	0	0.00	0;0	2
MCD	60	3	2.83	1.21;9.03	24	28.67	19.75;43.37	30	30.52	21.8;44.16	3	4.16	1.78;13.25	0	0.00	0;0	0
3-MCCD	40	11	10.39	6.14;19.2	13	15.53	9.52;27.32	7	7.12	3.78;15.38	6	8.32	4.25;19.1	2	23.95	9.28;96.53	1
MAD	8	1	0.94	0.33;6.13	3	3.58	1.53;11.42	4	4.07	1.87;11.19	0	0.00	0;0	0	0.00	0;0	0
MGA	6	1	0.94	0.33;6.13	4	4.78	2.2;13.14	1	1.02	0.35;6.61	0	0.00	0;0	0	0.00	0;0	0
HMGCLD	5	1	0.94	0.33;6.13	1	1.19	0.41;7.76	2	2.03	0.79;8.21	0	0.00	0;0	0	0.00	0;0	1

Abbreviations are the same as Table 2; ^a^Month-Old; ^b^Year-Old; ^c^A part of gender information of the dataset was missing and hence not available

For CD, 361 positive cases were detected in all age stages, and 94.74% of positive cases were detected in those who were less than 6 month-old. Amongst, 329 positive cases, i.e., 91.13% of all 361 positive cases, were from those who were 1 to 6 month-old. Apparently, the peak of CD was the period of 1 to 6 month-old (Table 2).

The data of OTCD are interesting for analysis. OTCD is an X-linked recessive urea cycle disorder (UCD) with a prevalence of 1 in 70,000–80,000 people [24]. According to a study [25], the onset of OTCD was also observed at later ages besides the early onset. Because OTCD follows the X-linked genetic pattern, male children were found to be more severe than female children. Another report showed that OTCD patients of female new-born infants had an incidence of 7% [26]. Our assays detected 190 positive cases of OTCD from 372,255 samples (Table 3). By gender, 129 positive cases were male and the rest 61 cases were female, which showed that males had significant higher OTCD positive case number (about twice) than that of females (Table 2). Our data showed that, all stages of ages had OTCD positive cases, but the distribution was not balanced. The two age stages with the highest positive case numbers of OTCD were less than (or equal to) 28 days and 7–36 month-old (Table 3). For those whose ages were less than or equal to 28 days, we found 66 OTCD positive cases which was 34.74% of all 190 OTCD positive cases. Interestingly, within the above 66 positive cases of OTCD, 62 cases were male and only 4 cases were female, which presented a large difference in positive case number. While for those who were older than 28 days, we had an OTCD positive case number of 124, where 67 cases were male, and 57 cases were female (Table 4). In such case, the difference of positive case number between male and female was less large than that of those whose ages were less than (or equal to) 28 days, though for those whose ages were older than 28 days, the positive case number of males was still slightly higher than that of females.

**Table 4:** OTCD positive case number distribution by gender and age stages

Gender	≤ 28 days	1–6 M.O.^a^	7–36 M.O.^a^	4–18 Y.O.^b^	> 18 Y.O.^b^	N.A.^c^
Male	62	3	28	31	3	2
Female	4	4	36	17	0	0

^a^Month-Old; ^b^ Year-Old;^c^ A part of age information of the dataset was missing and hence not available

### Provincial distribution of positive case rates

A rough observation suggested that different IMDs’ had different distribution patterns in China’s provinces. Using raw dataset, we calculated and analyzed also each IMD’s positive case numbers and positive rates of different provinces of China. In this section, we selected the top 5 IMDs with the highest number of positive case to further analyze and characterize their distribution patterns. The selected top 5 IMDs sorted from high to low positive case number are, MMA (3046), HPA (410), CD (361), PA (266) and GA-I (248) (Table 2).

**Fig. 2:**
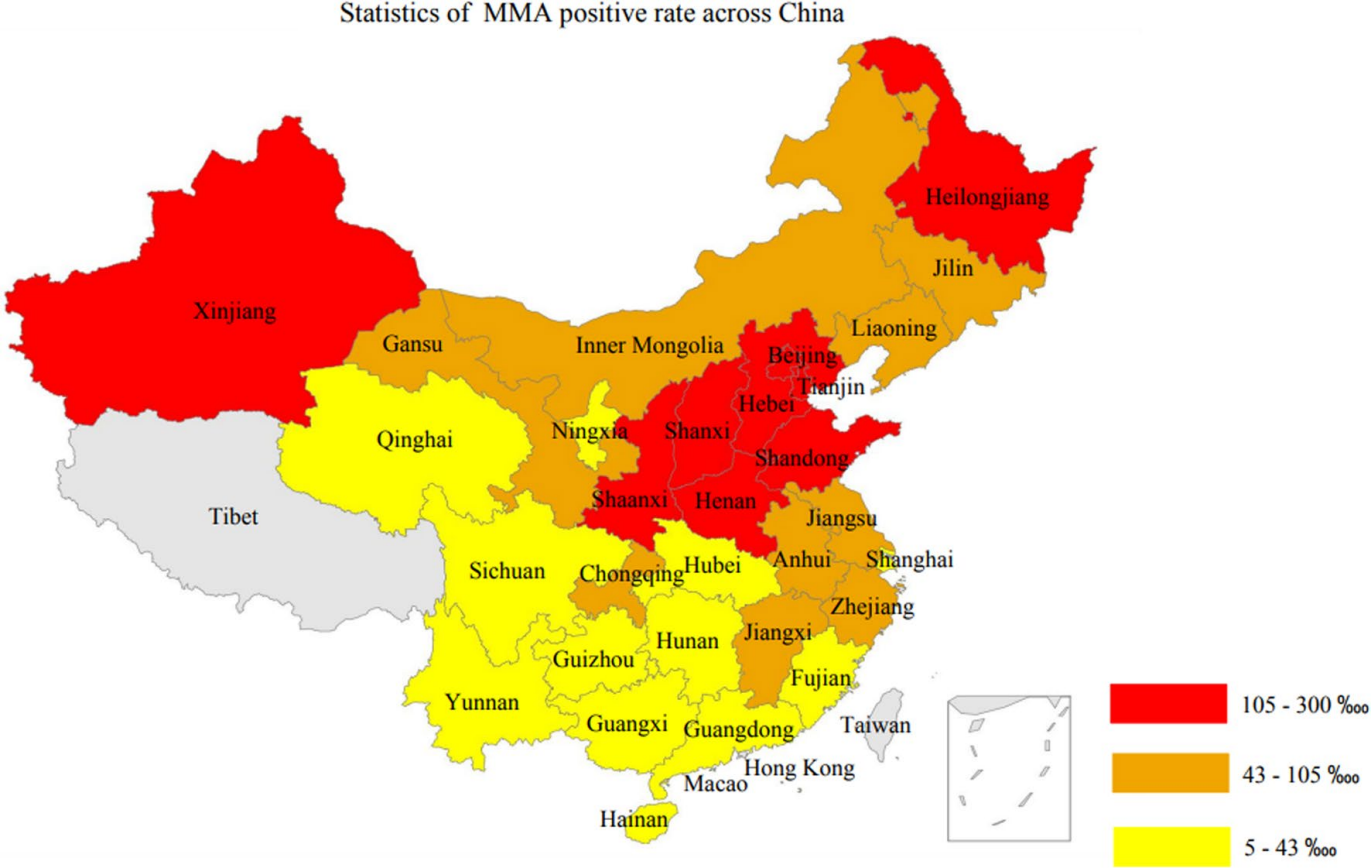
Distribution of MMA positive rates in different provinces of China

As seen in Table 1, the positive case number of MMA was significantly higher than other IMDs. For MMA itself, the provincial distribution rates ranked from high to low were Tianjin (296.30 ‱), Xinjiang (253.16 ‱), Shandong (233.27 ‱), Hebei (174.51 ‱), Heilongjiang (165.83 ‱), Shaanxi (141.43 ‱), Beijing (136.02 ‱), Henan (134.05 ‱), Shanxi (118.10 ‱), and Chongqing (105.11 ‱). And according to above rates, we mapped the provincial positive case rates and visualized the data into a China map (Fig. 2). Figure 2 indicated that MMA had a higher positive rate in North China than in South China in general. By region, we also calculated that, North China, East China, Northeast of China, Central China, Northwest of China, Southwest of China, and South China regions had MMA positive rates of 152.42 ‱, 127.10 ‱, 81.67 ‱, 78.39 ‱, 49.22 ‱, 37.28 ‱, and 19.95 ‱, respectively. Generally, the MMA positive rate of North China was the highest, and the number of the rate is 7.64 times higher than that of South China. Therefore, an imbalanced distribution pattern of positive rate of MMA was observed, which indicate that the incidence and prevalence might also distributed in an imbalanced way in China.

**Fig. 3:**
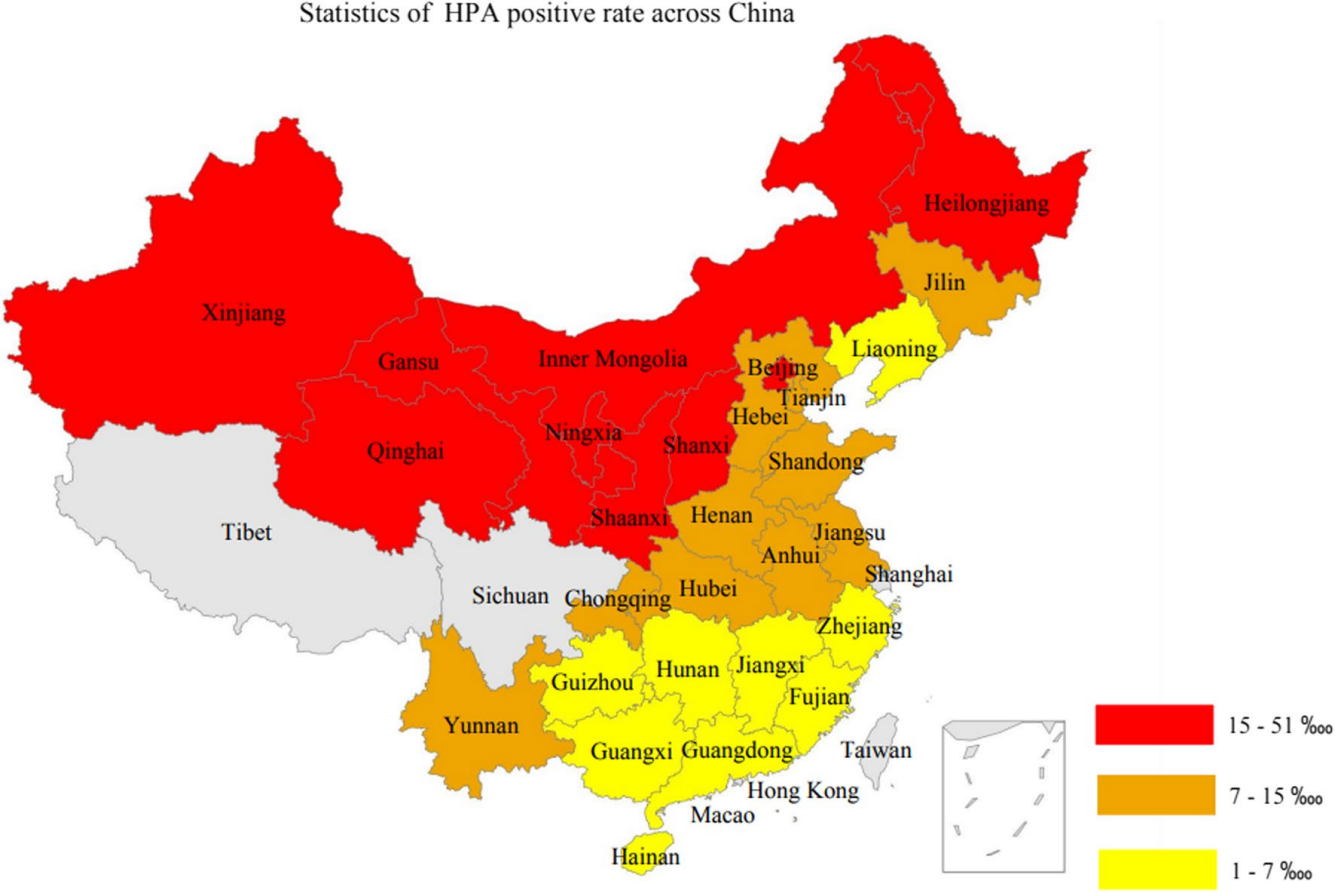
Distribution pattern of HPA positive rate in different provinces of China

Our analysis showed that, the top 10 provinces with the highest HPA positive rates were Qinghai (50.27 ‱), Shanxi (46.73 ‱), Gansu (45.18 ‱), Heilongjiang (43.26 ‱), Xinjiang (42.19 ‱), Ningxia (32.14 ‱), Shaanxi (30.75 ‱), Beijing (18.76 ‱), Inner Mongolia (15.84 ‱) and Yunnan (14.61 ‱). HPA is known to be an autosomal recessive disease. This IMD is more frequently seen in North China than in South China. Specifically, the Northwest area of China seems to have the highest incidence [27]. Among provinces aforementioned [27], five of them belong to the Northwest region of China. Therefore, our result is basically consistent with aforementioned report (Fig. 3).

**Fig. 4:**
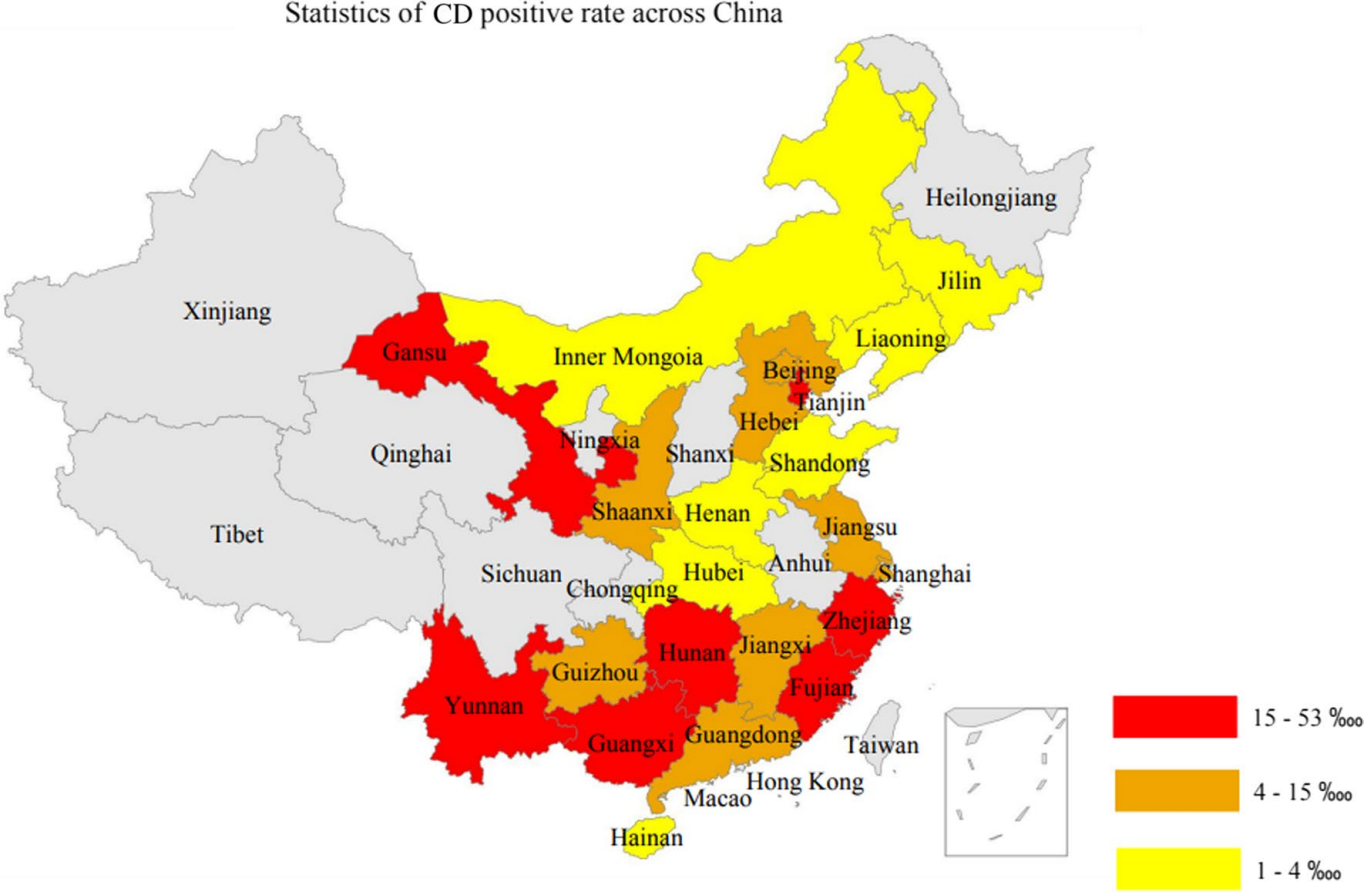
Distribution pattern of CD positive rate in different provinces of China

The same with HPA, CD is also a kind of autosomal recessive diseases. While in contrast to the regional distribution characteristics of HPA, our analysis showed that CD was more frequently seen in South China than in North China (Fig. 4). Our result showed that, the top 10 provinces with highest CD positive rates were Fujian (52.18 ‱), Zhejiang (36.35 ‱), Hunan (29.27 ‱), Yunnan (29.22 ‱), Guangxi (26.16 ‱), Tianjin (18.52 ‱), Gansu (15.06 ‱), Shanghai (14.56 ‱), Guizhou (12.82 ‱), and Guangdong (7.86 ‱). All these provinces are in South China except Gansu and Tianjin. We also found an interesting significant distribution difference divided by 30 degree north latitude. For Guangdong, Hunan, Fujian, Jiangxi, Yunnan, Guangxi, Hainan, Zhejiang, Guizhou, Anhui and Macao, whose latitudes are lower than 30 degree north latitude, 295 (81.72%) CD positive cases were identified amongst total 143,343 samples from above 11 provinces. While for the rest 21 provinces of China whose latitudes were higher than 30 degree north latitude, only 66 (18.28%) CD positive cases were identified from total 222,765 samples. The rough ratio of above percent numbers was close to 4:1. This evident difference is suggesting that, the prevalence and incidence of CD might be associated with the degree of latitude. At least in this work, China’s provinces with lower degree of latitude displayed a significant higher CD positive rate that those with higher degree of latitude.

**Fig. 5:**
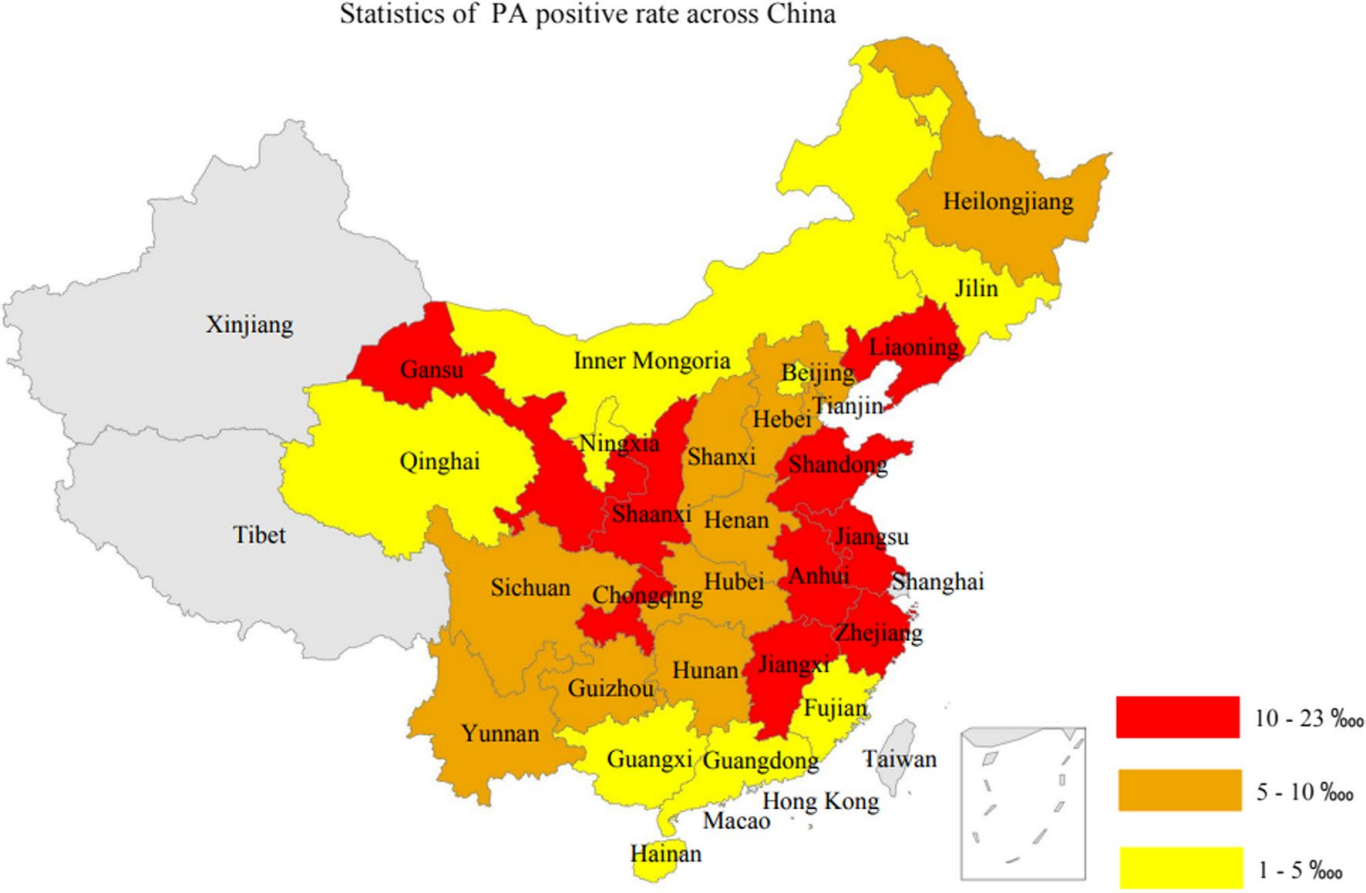
Distribution pattern of PA positive rate in different provinces of China

In our analysis, PA was the fourth IMD with high positive rate. Provinces with higher positive rates of PA were found to be Jiangxi, Zhejiang, Shaanxi, and Jiangsu (Fig. 5).

**Fig. 6:**
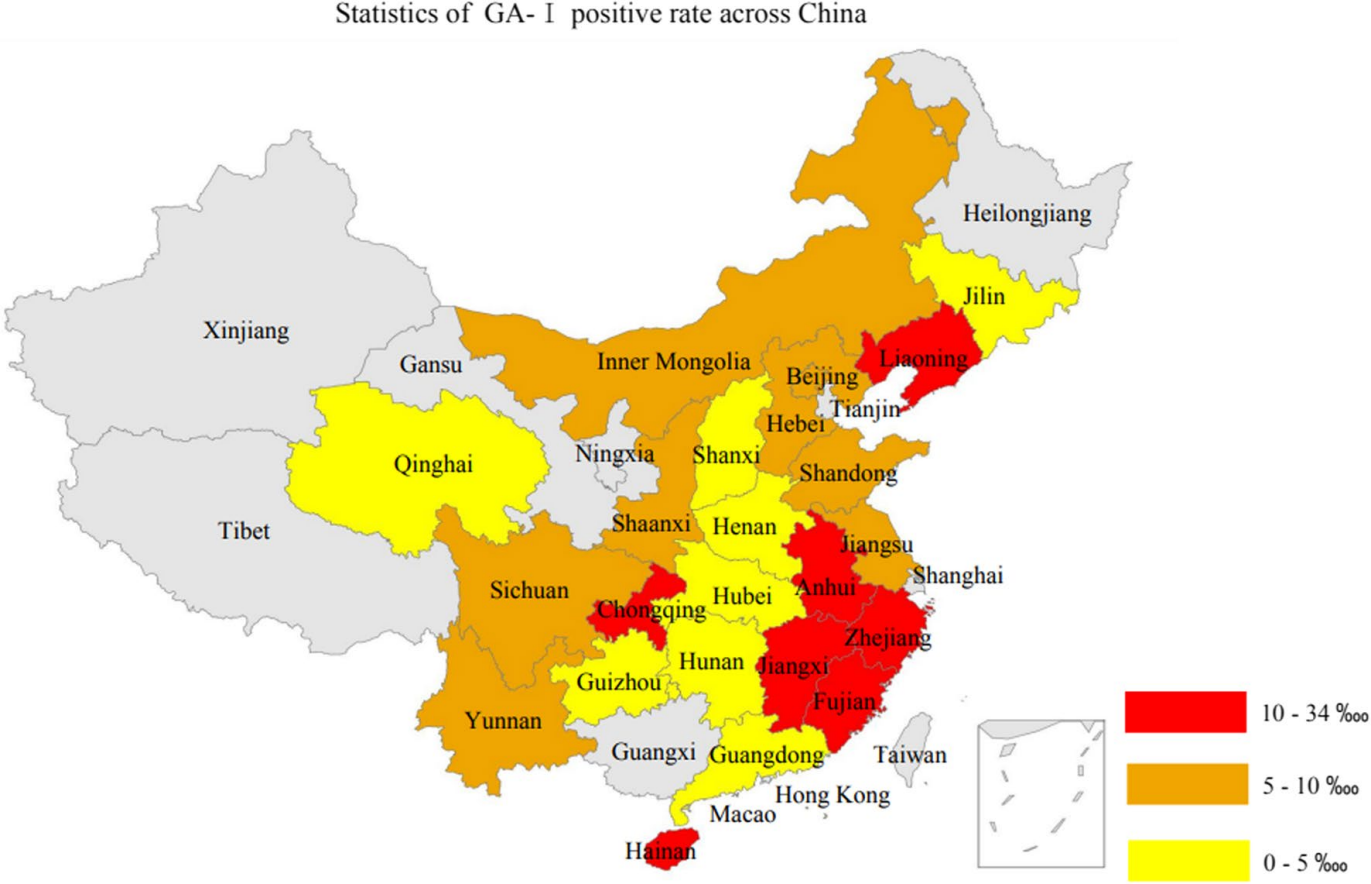
Distribution pattern of GA-I positive rate in different provinces of China

Figure 6 displayed the provincial distribution pattern of GA-I positive rate. The top 10 provinces with highest GA-1 positive rates were, Fujian, Anhui, Jiangxi, Chongqing, Zhejiang, Hainan, Liaoning, Beijing, Shandong, and Inner Mongolia. Amongst, Fujian had 33.32 ‱ GA-I positive rate, which was significantly higher that of Anhui, though Anhui’s GA-I positive rate (22.38 ‱) was the second highest. The third highest positive rate of GA-I was 18.17 ‱, from Jiangxi. By region and descending order, East China had GA-I positive rate of 13.37 ‱, North China had GA-I positive rate of 6.31 ‱, Southwest China had GA-I positive rate of 6.14 ‱, Northeast China had GA-I positive rate of 5.75 ‱, Central China GA-I positive rate of 4.77 ‱, South China had GA-I positive rate of 4.56 ‱; and Northwest China had GA-I positive rate of 1.81 ‱.

## Discussion

AKU is an autosomal recessive IMD. In theory, it affects both men and women in equal probability. Nonetheless, the disease was found to be more severe for men [28]. In our dataset, the number of positive case of AKU by gender (Male: Female) was 6:1 (Table 2), which is different from the theory. While another observation on 339 cases of AKU patients also reported an imbalanced ratio (about 2:1) of Male vs. Female number of positive AKU [29]. Unfortunately, the cause of the difference in disease severity is unknown so far.

So far, AKU is difficult to be diagnosed via public program of new-born screening of China because GC–MS measurement of urine homogentisic acid (which is the gold-standard diagnosis method for AKU) is currently not covered by the public screening program [28]. Another reason why AKU is hard to be diagnosis might be that, due to AKU has association with multiple types of clinical symptoms. Patients and clinicians may consider patient has other types of diseases rather than AKU [30]. Therefore, for people who have multiple types of symptoms, e.g., the arthritis and renal calculi/kidney stones, it is better not to forget to take an examination of AKU.

In result section, we mentioned that, after using 30 degree north latitude was used to divide China’s provinces, we observed provinces with lower latitude had significant higher positive rates of CD that those with higher latitude. Interestingly, another reports also compared positive CD data between Guangdong province and Shaanxi province, and stated that latitudinal gradient existed for CD, with a higher prevalence reported in lower latitudes [31, 32].We also analyzed the provincial latitudinal data and our own CD positive data. Nevertheless, we did not observe aforementioned latitudinal gradient in our dataset. At least, in our dataset, the positive rates of CD were not always increase along the increase of provinces’ degree number of latitude.

For clinical diagnosis, clinical signs and symptoms between MMA and PA are nonspecific, and hence it is not easy to distinguish between this pair of diseases [33]. Therefore, we suggested that there might be similarities between MMA and PA. Using the positive rate data of MMA and PA in different provinces in China, we conducted the Student’s test. As a result, the p-value was found to be smaller than 0.05, indicating that a significant difference existed between MMA’s and PA’s positive case provincial distribution. Therefore, in our dataset, we did not observe the association of distribution between MMA and PA. Moreover, a rough comparison between Figs. 2 and 5 could also support this conclusion.

The age ranges set for statistics become wider as the age increases. Most diseases show a trend of decreasing incidence rates with increasing age, indicating that IMDs usually occurs in childhood, except for some mild/late-onset IMDs such as OTCD and MMA. From a perspective of age, 1–6 months’ cases showed highest number of positive IMDs. i.e., 1441 cases. Age stage of younger than 28 days old was with the second highest number of IMDs positive. i.e., 1121 cases. High number of IMDs positive case of new-born infants might be because of the advancement and wider application of diagnostic technology based on tandem mass spectrometry and gas chromatography-mass spectrometry. Compared with DNA sequencing-based IMDs diagnosis, combination of LC–MS/MS and GC–MS for IMDs diagnosis generally takes shorter waiting time and is more economic. And thus the combination of LC–MS/MS and GC–MS technologies allow precise and quicker diagnosis of IMDs, which for IMDs patients, is the prerequisite of the life-saving early therapy.

Another reason why 1–6 months’ cases was peak age period might be that, several IMDs were failed to be screened in public programs for new-born screening. After the public screening program, children displayed abnormal symptoms found by parents and clinicians, and through IMDs clinical tests, children were confirmed to have IMDs.

We investigated the differences in IMDs incidence among provinces. The incidence distribution of most diseases in this study was consistent with previous studies. As aforementioned, the incidence of HPA is higher in northern China, mainly concentrated in northwestern regions such as Qinghai, Gansu, and Ningxia [34]. In contrast, CD showed a high incidence in southern China (Guangxi, Fujian) [31, 32] which might result from the heritability of IMDs. Most IMDs are autosomal recessive. And the primary cause of onset is whether the parents carry the pathogenic genes, so it is region-dependent and not affected by the environment. Furthermore, the study relying on Hospital Quality Monitoring System showed that one possible reason for the high incidence of MMA in Shanghai, Beijing, and Chongqing might be that patients are concentrated in these large cities with better medical resources [35]. Moreover, this study indicated that MMA concentrated in the eastern region because the major specimens were obtained from grassroots medical units in underdeveloped areas, which could reflect the practical disease distribution. Moreover, we did not find any previous studies reporting the incidence of GA-1, and our study indicated a high incidence of GA-1 in southeast China.

We also briefly reviewed the locations of hospitals that sent samples to KingMed. All samples of KingMed went through the commercial IMDs diagnosis program based on mass spectrometry, and the diagnosis program was paid by patients themselves. To make it simple, all samples of KingMed were 72% of the samples came from developed cities, and 8% samples were from poorer areas. This indicated that a significant difference of healthcare resource distribution across China. Another report also support this viewpoint [35]. Therefore, obviously, current policies and programs for healthcare have a lot to be improved. Current public new-born screening programs only cover limited types of IMDs diagnosis and the testing methods are immunoassays. Considered aforementioned advantages of gas chromotography and mass spectrometry technologies, and high IMDs positive cases and rates for infant who were younger than 6 months old, we recommend healthcare authorities to optimize healthcare policies based on data of our work. In light of aforementioned advantages of mass spectrometry-based IMDs diagnosis, e.g., lower costs, shorter waiting time, and coverage of diagnosis of more types of IMDs than immunoassay methods, we recommend that, the public new-born screening programs could include mass spectrometry-based assays for diagnosis of multiple types of IMDs. Also the screening programs would be better to cover infants who are less than 6 months old. Moreover, the programs should be spread to wider areas, especially those poorer areas with less healthcare resources.

There are several limitations of this works. First, a part of gender information is missing from the assay record due to the issue of database. Second, IMDs naturally have low prevalence compared with other types of common diseases. Consequently it is not easy to discover the patients (or positive cases) of IMDs. In our dataset, several types of IMDs had very low number of positive cases, making us difficult to further analyze and study these diseases. For example, the HMGCLD only had 5 positive cases and MGA only had 6 positive cases in our dataset. Thirdly, this work focused on organic acid disorders and a part of disorders of amino acid metabolism that, whose diagnostic indicators were associated with urine organic acids. Instead, this work did not included the analysis of fatty acid oxidation disorders and their relevant diagnostic indicators. Fourthly, since the samples in this research showed a geographical heterogeneity, and the uneven total number of cases in different regions, which might hurt representativeness of this study.

Though our dataset is large enough, above limitations limited us from doing further and deeper investigations on IMDs. While in the future, we plan to extend this work by overcoming above limitations. E.g., we could seek for other complementary resources so as to carry out further studies on IMDs. In addition, another large-scale dataset of LC–MS/MS-based China nationwide clinical blood testing for IMDs is also available from KingMed databases. We might integrate current dataset with that for further analysis, which might be able to depict better and more detailed global landscape of IMDs conditions in China. What is more, we might be able to explore and discovery better diagnostic indicators for IMDs, or use state-of-art artificial intelligence / machine learning methods to construct better and smarter diagnostic models.

## Conclusions

In this study, we collected and comprehensively analyzed 372,255 Chinese peoples’ clinical test data and IMDs diagnostic information (Table 2). Through statistical analyses, we characterized differences of IMDs’ distribution patterns by region, gender (Table 3), age etc. As a result, we discovered the unique distribution patterns of different IMDs. For example, the OTCD tended to progress on male infants who were less than 28 days old (Table 4). The MMA had the highest number of positive case among 16 types of IMDs (Table 2), and it had an imbalanced distribution pattern in China and its positive rate was significant higher in North China than South China (Fig. 2), and so on.

Results of our analyses provided most up-to-date information of IMDs of China and different provinces. Such information is valuable in all kinds of aspects. For instance, the provincial information informs domestic hospitals and clinicians about local status of IMDs. And provincial and nationwide information of IMDs also provide useful insights to the works of epidemiologists and workers of public health. Moreover, such information could also inspire medical policy makers via offering solid data and evidences for policy-making. Currently, regions of China are suffering from various kinds of medical and healthcare issues including but not limited to imbalanced medical resources distributions and insufficient budgets. Thus, we strongly recommend medical authorities of China to make better healthcare policies by referring to this comprehensive analytic study.

### Supplementary Information

**Additional file 1: Supplementary Table 1.** Assay metabolites.

**Additional file 2: Supplementary Table 2.** IMD diagnostic indicators.

## Data Availability

Raw data of this study contain sensitive information of patients. Due to legal and ethical consideration and restrictions, the raw data cannot be publicly shared. However, legal access of data is possible with a signed data access agreement or proposal.

## Abbreviations

IMDs: Inherited metabolic disorders
MS: Mass spectrometry
GC–MS: Gas chromotography coupled to mass spectrometry
LIMS: Laboratory information management system
AKU: Alkaptonuria
IVA: Isovaleric Acidemia
GA-I: Glutaric Acidemia Type I
MMA: Methylmalonic Acidemias
PA: Propionic Acidemia
CIT-I: Citrullinemia Type I
CD: Citrin deficiency disease
OTCD: Ornithine transcarbamylase deficiency
HPA: Hyperphenylalaninemia
MSUD: Maple syrup urine disease
TYR-I: Tyrosinemia type I
MCD: Multiple carboxylase deficiency
3-MCCD: 3-Methyl crotonyl-CoA carboxylase deficiency
MAD: Malonic acidemia deficiency
MGA: 3-Methylglutaconyl-CoA hydratase deficiency
HMGCLD: 3-Hydroxy-3-methylglutary-CoA lyase deficiency
