# Supplementary material for: China nationwide landscape of 16 types inherited metabolic disorders: a retrospective analysis on 372,255 clinical cases
Source: Orphanet J Rare Dis. 2023 Aug 3;18:228. doi: 10.1186/s13023-023-02834-y (PMC10398906; doi:10.1186/s13023-023-02834-y)
Supplement: Supplementary file 1 — Additional file 1: Supplementary Table 1. Assay metabolites. [file 13023_2023_2834_MOESM1_ESM.pdf]

Supplementary Table 1 Assay metabolites

ID	Organic acids	ID	Amino acids and acylcarnitines
1	Lactic acid	1	Alanine
2	2-hydroxy isobutyric acid	2	Aspartic acid
3	Hexanoic acid	3	Glutamic acid
4	Glycolic acid	4	Methionine
5	Oxalic acid	5	Phenylalanine
6	2-hydroxy butyric acid	6	Tyrosine
7	Glyoxylic acid	7	Leucine
8	3-hydroxy propionic acid	8	Tryptophan
9	Pyruvic acid	9	Valine
10	Valproic acid	10	Arginine
11	3-hydroxy butyric acid	11	Citrulline
12	3-hydroxy isobutyric acid	12	Glycine
13	2-hydroxy isovaleric acid	13	Ornithine
14	2-Methyl-3-hydroxy butyric acid	14	Glutamine
15	Malonic acid	15	Histidine
16	3-hydroxy isovaleric acid	16	Serine
17	2-Keto isovaleric acid	17	Threonine
18	Methylmalonic acid	18	Proline
19	Ethylhydracrylic acid	19	Free carnitine
20	Urea	20	Acetyl carnitine
21	4-hydroxy butyric acid	21	Propionyl carnitine
22	2-hydroxy isocaproic acid	22	Malonyl carnitine
23	3-hydroxy valeric acid	23	Butyryl carnitine
24	Acetoacetic acid	24	3-hydroxy butyryl carnitine
25	2-hydroxy-3-methyl valeric acid	25	Methylmalonyl carnitine
26	Benzoic acid	26	Isovaleryl carnitine
27	Octanoic acid	27	Tiglyl carnitine
28	2-Keto-3-methyl valeric acid	28	Glutaryl carnitine
29	2-Methyl-3-hydroxy valeric acid	29	3-hydroxy isovaleryl carnitine
30	Glycerol acid	30	Hexanoyl carnitine
31	Phosphoric acid	31	3-hydroxy hexanoyl carnitine
32	Ethylmalonic acid	32	Adipyl carnitine
33	2-Keto-isocaproic acid	33	Octanoyl carnitine
34	Acetylglycine acid	34	Octenoyl carnitine
35	Phenylacetic acid	35	Subaryl carnitine
36	Maleic acid	36	Decanoyl carnitine
37	Succinic acid	37	Decenoyl carnitine
38	Methylsuccinic acid	38	Decadienoyl carnitine
39	Glyceric acid	39	Sebacyl carnitine
40	Uracil	40	Dodecanoyl carnitine
41	Fumaric acid	41	Dodecenoyl carnitine
42	Propionylglycine	42	3-Hydroxydodecanoyl carnitine
43	Mevalonolactone	43	Myristoyl carnitine
44	Isobutyrylglycine	44	Myristoleyl carnitine
45	2-Propyl-3-hydroxy pentanoic acid	45	Tetradecadienoyl carnitine
46	Methylfumaric acid	46	3-hydroxy myristoyl carnitine
47	Glutaric acid	47	Palmitoyl carnitine
48	3-Methylglutaconic acid	48	Hexadecenoyl carnitine
49	3-Methylglutaric acid	49	3-hydroxy palmitoyl carnitine
50	2-Propyl-3-ketopentanoic acid	50	3-hydroxy palmitoleyl carnitine
51	2-Deoxytetronic acid	51	Octadecanoyl carnitine
52	Butyrylglycine	52	Octadecenoyl carnitine
53	Glutaconic acid	53	Linoleyl carnitine
54	Succinylacetone	54	3-hydroxy octadecanoyl carnitine
55	Decanoic acid	55	3-hydroxy octadecenoyl carnitine
56	2-Propyl-5-hydroxy pentanoic acid		

57	Isovalerylglycine		
58	Malic acid		
59	Adipic acid		
60	2-Hexenedioic acid		
61	5-Oxoproline		
62	3-methyladipic acid		
63	Thiodiglycolic acid		
64	2-Propyl-hydroxyglutaric acid		
65	7-hydroxy octanoic acid		
66	5-hydroxy-methyl-2-furoic acid		
67	Tiglylglycine		
68	3-Methylcrotonylglycine		
69	2-hydroxy glutaric acid		
70	3-hydroxy glutaric acid		
71	Phenyllactic acid		
72	Pimelic acid		
73	3-hydroxy-3-methylglutaric acid		
74	3-hydroxy phenylacetic acid		
75	2-Ketoglutaric acid		
76	4-hydroxy benzoic acid		
77	4-hydroxy phenylacetic acid		
78	Hexanoylglycine		
79	Phenylpyruvic acid		
80	N-Acetylaspartic acid		
81	2-hydroxy adipic acid		
82	Octenedioic acid		
83	3-hydroxy adipic acid		
84	Suberic acid		
85	2-Keto-adipic acid		
86	Aconitic acid		
87	Orotic acid		
88	Vanillic acid		
89	Homovanillic acid		
90	Azelaic acid		
91	Hipupuric acid		
92	Isocitric acid		
93	Citric acid		
94	Homogentisic acid		
95	Hippuric acid		
96	Methylcitric acid		
97	3-(3-hydroxy phenyl)-3-hydroxy propionic acid		
98	3-hydroxy octenedioic acid		
99	3-hydroxy suberic acid		
100	Vanilmandelic acid		
101	Sebacic acid		
102	Decadienedionic acid		
103	4-hydroxy phenyllactic acid		
104	4-hydroxy phenylpyruvic acid		
105	Indole-3-acetic acid		
106	Suberylglycine		
107	Palmitic acid		
108	2-hydroxy sebacic acid		
109	3-hydroxy sebacic acid		
110	2-hydroxy hippuric acid		
111	Dodecanedioic acid		
112	N-Acetyltyrosine		
113	Uric acid		
114	3,6-Epoxydodecanedioic acid		

115	3-hydroxy dodecanedioic acid		
116	3,6-Epoxytetradecanedioic acid		
