# Supplementary material for: China nationwide landscape of 16 types inherited metabolic disorders: a retrospective analysis on 372,255 clinical cases
Source: Orphanet J Rare Dis. 2023 Aug 3;18:228. doi: 10.1186/s13023-023-02834-y (PMC10398906; doi:10.1186/s13023-023-02834-y)
Supplement: Supplementary file 2 — Additional file 2: Supplementary Table 2. IMD diagnostic indicators. [file 13023_2023_2834_MOESM2_ESM.pdf]

Supplementary Table 2 IMD diagnostic indicators

IMD	Organic acid indicators for IMD diagnosis	Amino acids and acylcarnitines indicators for IMD diagnosis
Amino acid disorders		
HPA	Phenylpyruvic acid; Phenylacetic acid; Phenyllactic acid ;	Phenylalanine;Tyrosine
CD	4-hydroxy phenyllactic acid; 4-hydroxy phenylpyruvic acid;	Citrulline; Methionine;
OTCD	Orotic acid; Uracil;	Citrulline
MUSD	2-hydroxy isovaleric acid; 2-Keto-isovaleric acid; 2-Keto-3-methylvaleric acid; 2-Keto-isocaproic acid;	Leucine; Valine
CIT-I	Orotic acid; Uracil;	Citrulline
AKU	Homogentisic acid;	/
TYR-I	Succinylacetone;	Tyrosine
Organic acidemias		
MMA	Methylmalonic acid; Methylcitric acid;	Propionyl carnitine;Acetyl carnitine
PA	3-hydroxy propionic acid; Methylcitric acid;	Propionyl carnitine;Acetyl carnitine
GA-I	Glutaric acid; Glutaconic acid; 3-hydroxy glutaric acid;	Glutaryl carnitine
IVA	Isovalerylglycine;	Isovaleryl carnitine
MCD	Tiglylglycine; 3-Methylcrotonylglycine; 3-hydroxy isovaleric acid; 3-hydroxy propionic acid; Methylcitric acid;	3-hydroxy isovaleryl carnitine
3-MCCD	Tiglylglycine; 3-Methylcrotonylglycine; 3-hydroxy isovaleric acid;	3-hydroxy isovaleryl carnitine
MAD	Malonic acid;	Malonyl carnitine
MGA	3-Methylglutaconic acid;	3-hydroxy isovaleryl carnitine
HMGCLD	3-hydroxy-3-methylglutaric acid;	3-hydroxy isovaleryl carnitine

HPA: Hyperphenylalaninemia; CD: Citrin Deficiency Disease; OTCD: Ornithine Transcarbamylase Deficiency; MSUD: Maple Syrup Urine Disease; CIT-I: Citrullinemia Type I; AKU: Alkaptonuria; TYR-I:Tyrosinemia type I; MMA: Methylmalonic Acidemias; PA: Propionic Acidemia; GA-I: Glutaric Acidemia Type I; IVA: Isovaleric Acidemia; MCD: Multiple Carboxylase Deficiency; 3-MCCD: 3-Methyl Crotonyl-CoA Carboxylase Deficiency; MAD: Malonic Acidemia Deficiency;MGA:3-Methylglutaconyl-CoA Hydratase Deficiency; HMGCLD: 3-Hydroxy-3-Methylglutary-CoA Lyase Deficiency
